# The Importance of Diagnostic Testing during a Viral Pandemic: Early Lessons from Novel Coronavirus Disease (COVID-19)

**DOI:** 10.4269/ajtmh.20-0216

**Published:** 2020-03-30

**Authors:** Philip J. Rosenthal

**Affiliations:** Department of Medicine, University of California, San Francisco, California

At the time of this writing (late March 2020), the pandemic of novel coronavirus disease (COVID-19) is rapidly expanding across much of the world, as it wanes in parts of Asia. Public health authorities and healthcare workers are struggling to appropriately manage potentially infectious individuals to limit transmission to others and to appropriately care for those ill with proven or suspected COVID-19. Approaches have varied from country to country, with varied success in controlling local epidemics. Many lessons are being learned. One lesson is the value of readily available and prompt diagnostic testing for the virus.

The COVID-19 pandemic emerged in the city of Wuhan, Hubei Province, China, in December 2019. In late December, evaluation of bronchoalveolar lavage fluid from an ill patient identified a betacoronavirus, and the virus, named severe acute respiratory syndrome coronavirus-2 (SARS-CoV-2), was sequenced by early January 2020, enabling rapid development of molecular testing.^1^ Standard testing for acute infection entails reverse transcription polymerase chain reaction (RT-PCR) amplification of reverse-transcribed viral RNA from respiratory specimens, most commonly nasopharyngeal swabs, but also oropharyngeal swabs, sputum, and bronchoalveolar lavage fluid.^2^ Antigen-based and serological testing for SARS-CoV-2 infection are under study. Antigen-based tests may suffer from limited sensitivity, as seen for related viruses.^2^ Serological testing, once standardized, will be valuable for epidemiological studies evaluating the extent of the pandemic, but may be limited for acute diagnosis.

Two critical features of a successful response to a pandemic respiratory virus are early detection and isolation of potentially infectious individuals. In China, where the pandemic began, initial delays in action contributed to the rapid growth of the epidemic, but acknowledgment of the outbreak in late December 2019 was followed by development of a molecular test for the infection within about 2 weeks. The rapid development of a reliable diagnostic was of great value. Availability of diagnostics was initially limited, but the establishment of extreme isolation for Hubei Province likely played a major role in allowing the Chinese epidemic, although very large, to be controlled over some weeks. Epidemic responses were different in other countries in Asia, including South Korea, Singapore, and Taiwan. In these countries, the early response was marked by wide access to molecular testing, benefitting from the earlier advances in China. Widespread testing of those with suggestive symptoms of SARS-CoV-2 infection or contact with a patient, followed by aggressive contact tracing, allowed for isolation of those infected and quarantine of contacts. In these countries, COVID-19 outbreaks were controlled reasonably quickly, with relatively few deaths compared to some other countries with similar numbers of reported infections.

Around the world, COVID-19 responses have varied greatly. In particular, widespread viral testing has been much more available in some countries than others. In the United States, to date, tests have been in limited supply, with testing prioritized to hospitalized patients with respiratory disease, symptomatic healthcare workers, and contacts of known cases of COVID-19.

In a case report published in this issue of the *Journal*, a 56-year-old woman who traveled from the epicenter of the pandemic in Wuhan, China, to Thailand in late January 2020 presented 8 days after her husband was diagnosed with COVID-19 without symptoms but with concern for infection.^3^ She had been tested 4 days earlier by RT-PCR for SARS-CoV-2 of nasopharyngeal and throat swabs, and results were negative. On the day of admission, she denied symptoms and was afebrile with normal oxygenation, but coughing was noted during examination. Chest X-ray showed an alveolar opacity suggestive of COVID-19, and the patient was admitted, with respiratory isolation. Repeat RT-PCR testing for SARS-CoV-2 of sputum on the day of admission was deemed inconclusive. The patient reported sore throat, mild cough, and diarrhea 2 days after admission. A third RT-PCR test, of sputum, 3 days after admission, was positive for SARS-CoV-2. The patient’s symptoms and chest X-ray infiltrates worsened, and chest computed tomography and ultrasound were abnormal, but findings subsequently improved, and the patient was discharged after two negative SARS-CoV-2 tests.

The case report from Thailand demonstrated prompt and appropriate management of a patient with exposure to SARS-CoV-2 and then diagnosis facilitated by repeat RT-PCR testing and lung imaging. This diagnosis facilitated appropriate management through the course of SARS-CoV-2 infection. Early isolation is important, as COVID-19 may be highly infectious in asymptomatic individuals or before symptoms are apparent.^4,5^ Notably, this diagnosis would likely have been missed in the United States, where, to date, clinicians struggle to provide diagnostic tests. The Thai patient would have fit some current criteria for testing in the United States, as she was a contact of a patient with documented infection, but U.S. guidelines do not include repeat testing in the event of initial negative tests. This is unfortunate, as it appears that COVID-19 patients commonly have negative tests early in the course of illness, at times with abnormal chest imaging, as in the described case report and in a report from China,^6^ but the limited supply of tests necessitates restrictive testing algorithms that do not incorporate repeat testing. By contrast, the patient described in the case report received appropriate isolation and then appropriate discontinuation of isolation, based on a total of 5 RT-PCR tests.

Moving forward, as we work to control the COVID-19 pandemic and as we plan for future pandemics, a key lesson is that early availability of diagnostic testing is of great value for patient management and public health. Thus, the development, validation, scale-up in manufacture, and distribution of diagnostic tests should be of highest priority in early preparation during an emerging infectious disease outbreak.
